# Aspects écho-cardiographiques au cours de la drépanocytose en Guadeloupe

**DOI:** 10.11604/pamj.2014.18.45.3820

**Published:** 2014-05-13

**Authors:** Louis Igor Ondze-Kafata, Alain Sanouiller, Mona Hedreville, Segho Hedreville, Laurent Larifla

**Affiliations:** 1 Service de Cardiologie et Médecine Interne, CHU de Brazzaville, Brazzaville, Congo; 2 Centre caribéen de la drépanocytose, CHU de Pointe-à-Pitre/ Abymes, Pointe-à-Pitre/ Abymes, Guadeloupe; 3 Service de Cardiologie, CHU de Pointe-à-Pitre/ Abymes, Pointe-à-Pitre/ Abymes, Guadeloupe

**Keywords:** Drépanocytose, échocardiographie, hypertension artérielle pulmonaire, sickle cell disease, echocardiography, pulmonary hypertension

## Abstract

**Introduction:**

L'atteinte cardiovasculaire représente le facteur pronostique majeur de la drépanocytose.

**Méthodes:**

C’était une étude transversale et descriptive dans le service de cardiologie du centre hospitalier et universitaire de Pointe-à-Pitre.

**Résultats:**

Nous avons inclus 82 patients, 23 hommes (28%) et 59 femmes (72%), âgés en moyenne de 40,0±12 ans. On avait 39 patients porteurs d'hémoglobinose SS (49,4%) et 40 porteurs d'hémoglobinose SC (50,6%). Le taux d'hémoglobine de base était en moyenne de 9,8±1,8 g/dl et celui de l'hémoglobine fœtale était en moyenne de 5,5±5,3 g/dl. La fraction de raccourcissement était de 36±4,9% en moyenne, la FeVG moyenne était de 65±5,9%. On avait 68 patients avec un profil mitral normal (82,9%) et 14 patients avec un profil pseudo normal (17,1%). On avait une dilatation ventriculaire gauche chez 26 patients (31,7%), ventriculaire droite chez 14 patients (17.1%), une dilatation auriculaire gauche chez 36 patients (43.9%), auriculaire droite chez 11 patients (13.4%). On avait une hypertrophie ventriculaire gauche chez 13 patients soit 15.9%. L'hypertension artérielle pulmonaire a été retrouvée chez 43 patients soit 52,4%. Après régression logistique, les facteurs suivants ont été associés à la présence d'une HTAP: âge (p=0,0024), le taux hémoglobine de base (p=0,032), le taux d'hémoglobine fœtale (p=0,011), le type SS (p=0,040), la dilatation de l'oreillette gauche (p=0,0012) et la dilatation de ventricule droit (p=0,047). Après comparaison, L'hémoglobinose SS s’était avérée plus sévère.

**Conclusion:**

La drépanocytose est une pathologie sévère dont le pronostic est aggravé par l'atteinte cardiaque.

## Introduction

La drépanocytose est une maladie génétique autosomique récessive secondaire à une mutation ponctuelle au niveau du sixième codon du gène de la β-globine avec substitution de l'adénine par la thymine sur le chromosome 11 [1]. Elle touche plus de 50 millions de personnes dans le monde en particulier en Afrique noire et le pourtour méditerranéen. En Guadeloupe, elle touche un nouveau-né sur 260 [2]. La littérature abonde d’études sur le cœur du drépanocytaire [3–5], mais nous disposons à ce jour de peu d’études sur les patients drépanocytaires guadeloupéens. Aussi avons-nous entrepris ce travail avec pour objectifs de rapporter les aspects échographiques du cœur des drépanocytaires guadeloupéens, d'identifier les différentes anomalies éventuelles en particulier la présence de l'hypertension artérielle pulmonaire et d'en rechercher les facteurs associés.

## Méthodes

Il s′agit d′une étude transversale descriptive qui s′était déroulée sur une période de quatre mois (1er janvier 2010 au 30 avril 2010) dans le service de cardiologie du centre hospitalier et universitaire de Pointe-à-Pitre en Guadeloupe. Après accord du comité d’éthique de l'hôpital, nous avons inclus par tirage au sort du fichier du centre caribéen de la drépanocytose en Guadeloupe des patients âgés d'au moins 16 ans, suivis régulièrement au centre caribéen de drépanocytose de la Guadeloupe, porteurs d'un syndrome drépanocytaire majeur type SS ou SC. Pour des raisons logistiques et budgétaires, le nombre des patients a été limité à 82. Leur consentement éclairé a été ensuite obtenu sur un formulaire approprié. Nous avons exclu les femmes enceintes, les mineurs de moins de 16 ans et les hétérozygote AS ou AC.

Les données biologiques suivantes ont été obtenues à partir des dossiers médicaux: type d'hémoglobinopathie, taux d'hémoglobine de base, taux hémoglobine fœtale, taux de LDH et l'existence d'un déficit en G6PD associé.

Tous les patients ont bénéficié d'une échographie cardiaque transthoracique sur un appareil Philips IE33 doté d'une sonde 3.5 Mhz et de la technologie Doppler. L’échographie TM et bidimensionnelle a permis la mesure selon les recommandations de l'American Society of Echocardiography (ASE) [6] les dimensions de la racine aortique, de l'oreillette gauche, du ventricule gauche, du ventricule droit, des surfaces de l'oreillette gauche et droite, de la fraction de raccourcissement, de la fraction d’éjection par la méthode de Teicholtz et celle de Simpson, ainsi que de la masse du ventricule gauche. Le doppler pulsé a permis de mesurer l'onde E, l'onde A, et le temps de décélération du flux mitral ainsi que l'onde S, l'onde D, l'onde A du flux veineux pulmonaire. La vitesse de propagation a été réalisée en mode TM couleur avec la vitesse d'aliasing à 75% de l'amplitude de l'onde E mitrale. L'onde Ea à l'anneau mitral a été obtenue en doppler tissulaire sur la paroi latérale. Enfin, la pression artérielle pulmonaire a été évaluée par doppler continu sur la vitesse maximale de la fuite tricuspidienne. Nous avons considéré le ventricule gauche comme dilaté lorsque son diamètre télé diastolique indexé à la surface corporelle était supérieur à 32mm/m2 chez la femme et 31mm/m2 chez l'homme. L'oreillette gauche a été considérée comme dilatée lorsque sa surface était supérieure à 20 cm^2^. L'hypertrophie ventriculaire gauche été définie par une masse ventriculaire gauche indexée supérieure à 115g/m2 chez l'homme et à 95 g/m2 chez la femme en l'absence de dilatation du ventricule gauche. La dysfonction systolique du ventricule gauche a été définie par une fraction d’éjection ventriculaire gauche inférieure à 55%. L'analyse de la fonction diastolique au doppler pulsé et tissulaire à l'anneau mitral a permis de regrouper les patients en trois groupes [7]: trouble de la relaxation, trouble de la compliance, et profil pseudo normal. L'hypertension artérielle pulmonaire (HTAP) a été définie par une vitesse maximale de la fuite tricuspidienne supérieure ou égale à 2,5m/s [8, 9].

### Patients

Les 82 patients se répartissaient en 59 femmes (72%), et 23 hommes (28%) âgés en moyenne de 40,0±12 ans avec des extrêmes de 16 à 70 ans. Dans 39 cas (49,4%) les patients étaient porteurs d'hémoglobinose SS, et dans 40 soit 50,6% d'hémoglobinose SC (50,6%). Un déficit en G6PD était associé chez six patients. Le taux d'hémoglobine de base était en moyenne de 9,8±1,8 g/dl avec des extrêmes de 6 à 13,5. Le taux d'hémoglobine fœtale était en moyenne de 5,5±5,3 g/dl avec des extrêmes de 0,4 à 20,6. Le taux des LDH était en moyenne de 392,6±33,9 UI/L avec des extrêmes de 100 à 2750.

### Analyse statistique

Les données ont été saisies avec le logiciel Microsoft Excel 2007 et analysées avec le logiciel SPSS version 13.0. Les variables quantitatives ont été exprimées sous forme de moyenne et écart type, et les variables qualitatives sous forme de pourcentage. Le test de Kruskal-Wallis a été utilisé pour comparer plusieurs échantillons indépendants. Une analyse multi variée a été pratiquée pour identifier les facteurs associés à l'hypertension artérielle pulmonaire. Le seuil de signification était fixé à p inférieur ou égal à 0,05.

## Résultats

Les dimensions des différentes cavités cardiaques sont indiquées dans le Tableau 1. Nous avons observé une dilatation ventriculaire gauche chez 26 patients (31,7%), ventriculaire droite chez 14 patients (17,1%), une dilatation auriculaire gauche chez 36 patients (43,9%), auriculaire droite chez 11 patients (13,4%). La fraction de raccourcissement était en moyenne de 36±4,9% avec des extrêmes de 26 à 45%, la fraction d’éjection était en moyenne de 65±5,9% avec des extrêmes de 50 à 80% par la méthode de Teicholtz et par celle de Simpson. Seuls trois patients avaient présenté une dysfonction systolique modérée avec une fraction d’éjection ventriculaire gauche entre 50 et 55%. Une hypertrophie ventriculaire gauche existait chez 13 patients soit 15,9%.

**Tableau 1:** Dimensions des différentes cavités cardiaques de 82 patients drépanocytaires

	Moyenne (ET)	Extrêmes	Dilatations
			HVG
RAO (mm)	27,5 (4,04)	18 - 39	1(1.2)
DOG (mm)	36,16 (6,74)	17 - 56	18(22.0)
SOG (cm^2^)	20,87 (6,61)	10 - 48,6	36(43.9)
SOD (cm^2^)	15,16 (4,7)	6,7 - 29,9	11(13.4)
DVD (mm)	20,5 (6,44)	8 - 40	14(17.1)
DTDVG (mm)	50,63 (6,14)	32 - 63	26(31.7)
DTSVG (mm)	32,2 (4,48)	22 - 43	-
SIV (mm)	9,13 (1,83)	5 - 13	-
PP (mm)	9,04 (1,48)	6 - 13	-
MASSE VG(g)	165,59 (51,58)	81,7 - 321	13(15.9)

RAO : racine aortique ; DOG : diamètre de l'oreillette gauche ; SOG : surface de l'oreillette gauche ; SOD : surface de l'oreillette droite ; DVD : diamètre du ventricule droit ; DTDVG : diamètre télédiatolique du ventricule gauche ; DTSVG : diamètre télé systolique du ventricule gauche ; SIV : septum inter auriculaire ; PP : paroi postérieure ; VG : ventricule gauche

Les données sur la fonction diastolique sont résumées dans le Tableau 2. Dans 68 cas soit 82,9%, les patients avaient un profil normal et dans 14 cas soit 17,1% un profil pseudo normal. Une hypertension artérielle pulmonaire a été retrouvée chez 43 patients soit 52,4%.

**Tableau 2:** Données relatives à la fonction diastolique de 82 patients drépanocytaires

	Population totale	
	Moyenne (ET)	Extrêmes
Onde E (cm/s)	95,24 (19,17)	58 - 135
Onde A (cm/s)	70,45 (18,68)	40 - 120
Rapport E/A	1,45 (0,48)	0,7 - 3,3
Durée A(ms)	136,64 (2,41)	99 - 225
Temps de décélération onde E (ms)	151,1 (41,47)	67 - 254
Onde S (cm/s)	61,82 (3,66)	21 - 91
Onde D (cm/s)	53,42 (13,97)	25,2 - 83
Rapport S/D	1,25 (0,41)	0,6 - 2,7
Onde Ea (cm/s)	13.76(3.52)	5 – 20.9
Vitesse de propagation (cm/s)	89,66 (4,28)	38 - 115
Rapport E/Ea	7,41 (2,22)	4,1 - 15,8
Rapport E/Vp	1,24 (0,47)	0,2 - 2,8

Après régression logistique (Tableau 3), les facteurs suivants ont été associés à la présence d'une HTAP: l’âge (p=*0,0024*), le taux hémoglobine de base (p=*0,032*), le taux d'hémoglobine fœtale (p=*0,011*), le type SS d'hémoglobine (p=*0,040*), la dilatation de l'oreillette gauche (p=*0,0012*) et la dilatation de ventricule droit (p=*0,04. 7*).

**Tableau 3:** Facteurs associés à HTAP chez 82 patients drépanocytaires

	HTAP(+)	HTAP(-)	
	(n=43)	(n =39)	p
	N(%) ou Moy(ET)	N(%) ou Moy(ET)	
**Données clinico-biologiques**			
Age (an)	43,0 (11,8)	37,0 (11,5)	0.0024
Sexe (hommes)	13,0 (30,2)	10,0 (25,6)	0.640
Taux d'hémoglobine de base /dl)	9.44 (1.84)	10.35 (1.80)	0.032
Taux d'hémoglobine fœtale (%)	3.86 (3.37)	6.97 (5.7)	0.011
LDH (UI)	385.25 (200.39)	400 (426.16)	0.327
Type SS d'hémoglobine	26 (65%)	13 (33%)	0.040
**Données échographiques**			
DTDVG (mm)	51.86 (5.7)	49.28 (6.38)	0.057
DVD (mm)	21.84 (6.87)	19.03 (5.65)	0.047
DOG (mm)	37.44 (6.5)	34.77 (6.78)	0.068
SOG (cm^2^)	23.07 (6.95)	18.45 (5.3)	0.0012
SOD (cm^2^)	15.70 (5.4)	15.6 (3.8)	0.518
FeVG Simpson (%)	65.5 (5.53)	65.26 (7.15)	0.518

DOG : diamètre de l'oreillette gauche ; SOG : surface de l'oreillette gauche ; SOD : surface de l'oreillette droite ; DVD : diamètre du ventricule droit ; DTDVG : diamètre télédiatolique du ventricule gauche ; FeVG : fonction d’éjection du ventricule gauche.

Les données sur la comparaison entre les deux types d'hémoglobinopathie sont résumées dans le Tableau 4. L'hémoglobinose SS était associée à un taux d'hémoglobine et d'hémoglobine fœtale plus bas (p10, 11]. Dans cette forme hétérozygote SC, l’âge de la manifestation clinique de la maladie était plus tardif, l'hémoglobine de base plus élevée, les signes cliniques plus atténués [12], les cavités cardiaques moins dilatées [13], l'HTAP est moins fréquente[10], la durée du recours à l'oxygénothérapie en cas de syndrome douloureux thoracique aigu plus courte [14]. Nous avons confirmé ces données: en effet, nous avons observé, en comparant ces deux formes d'hémoglobinopathie, une atteinte moins sévère dans la forme hétérozygote composite SC avec des taux d'hémoglobine de base moins bas et d'hémoglobine fœtale plus élevés, des taux de LDH moins élevés, des cavités cardiaques moins dilatées et l'HTAP moins fréquente que dans la forme homozygote SS.

**Tableau 4:** Comparaison des données cliniques et biologiques, et écho cardiographiques des drépanocytaires SS (n=39) et SC (n=40)

	SS	SC	
	N(%) ou Moy(ET)	N(%) ou Moy(ET)	p
**Données clinico-biologiques**			
Age (an)	38 (11,4)	42,1 (12,37)	0,118
Sexe (hommes)	11 (28,2)	9.00 (22,5)	0,56
Hb base (g/dl)	8,47 (1,41)	11,24 (1,12)	0,001
Hb fœtale (%)	2.05 (1.86)	8,77 (5,51)	0,001
LDH (UI)	527 (25,47)	265 (100,77)	0,001
**Données échographiques**			
HTAP	26(67%)	14,0 (36%)	0,004
DTDVG (mm)	53,26 (5,17)	47,43 (5,37)	0,001
SOG (cm^2^)	23,75 (7,1)	17,98 (4,9)	0,001
SOD (cm^2^)	16,12 (5,24)	14,03 (4,03)	0,049
FeVG Simpson (%)	1(2,6%)	2,00 (5,0%)	0,57
Trouble de la relaxation	8(20,5)	6,00 (15%)	0,52
RAO	28,26 (3,49)	26,74 (4,4)	0,155

SOG : surface de l'oreillette gauche ; SOD : surface de l'oreillette droite ; DTDVG: diamètre télédiatolique du ventricule gauche ; FeVG : fonction d’éjection du ventricule gauche ; RAO : racine aortique ; HTAP : hypertension artérielle pulmonaire

### Dimensions des différentes cavités

Dans ce travail, nous avons retrouvé une dilatation de toutes les cavités cardiaques dans des proportions variables, en particulier l'oreillette gauche (43,9%) et le ventricule gauche (31,7%). Ces résultats concordent avec les données de la littérature [3, 15–17]. Eddine et al[4] avaient montré que la dilatation de ventricule gauche ainsi que l'augmentation de la masse ventriculaire gauche était corrélée à l'anémie et non modifiée par le traitement par l'hydroxyurée. Mais cette corrélation n'a pas été retrouvée par d'autres [18]. La masse ventriculaire gauche était augmentée chez 15,9% de nos patients. Cette hypertrophie ventriculaire gauche a été rapportée dans des proportions de 46% par Johnson et al [19]. Ces auteurs ont observé une association avec une désaturation nocturne et à la marche sans rapport avec une hypertension artérielle pulmonaire. Taksande et al[20] avaient retrouvé en plus une dilatation de la racine aortique, ce qui n’était retrouvé que dans un cas dans notre travail.

### Fonction systolique du ventricule gauche

La fonction systolique du ventricule gauche était normale dans la grande majorité de nos cas. La majorité des travaux de la littérature [3, 4] n'avaient pas retrouvé de dysfonction systolique en terme de fraction d’éjection du ventricule gauche ou de fraction de raccourcissement entre les patients drépanocytaires et non drépanocytaires. Certains auteurs [16, 21] ont rapporté une hyperkinésie, probablement en rapport avec l'anémie.

Cependant, en recourant non pas aux seules fraction de raccourcissement et fraction d’éjection mais à des paramètres plus performants comme l'indice de Tei, on pouvait mettre en évidence plus précocement des stigmates de dysfonction systolique chez les patients drépanocytaires [3, 22].

### Fonction diastolique du ventricule gauche

Dans notre étude, 14 patients soit 17,1% présentaient un trouble de la relaxation au doppler. Certains auteurs [5, 20] n'ont certes pas retrouvé de différences significatives entre drépanocytaires et non drépanocytaire en terme de fonction diastolique, mais certaines anomalies ont été rapportées surtout chez les patients très anémiques ayant nécessité des transfusions itératives. Ainsi, la dysfonction diastolique était affirmée chez le drépanocytaire par un temps de décélération de l'onde E mitrale augmenté [22], par un rapport E/Ea supérieur à 8 [4], et par un temps de remplissage du ventricule gauche raccourci avec une grande onde A de remplissage rapide[17]. La fonction diastolique chez les drépanocytaires a déjà été étudiée par plusieurs auteurs [23] qui avaient montré que la dysfonction diastolique était liée à la sévérité de la maladie et non à la surcharge en fer secondaire aux transfusions itératives.

### Hypertension artérielle pulmonaire et drépanocytose

L'HTAP est une complication classique de la drépanocytose [9, 24–28]. Nous l'avons retrouvée chez 52,4% des patients âgés de 16 à 70 ans (en moyenne de 40,0±12 ans). Cette proportion était de 11 à 14% chez l'enfant [27], et de 25 à 50% chez l'adulte [8, 9, 24–26, 28, 29]. Elle est un facteur pronostic majeur en terme de mortalité et de morbidité [9, 24–28]. Sa présence aggrave le pronostic avec un risque de mortalité à deux ans entre 40 et 50% [15]. Nous avons observé qu'elle était associée à la dilatation du ventricule droit et de l'oreillette gauche. On sait [29, 30] que l’ HTAP était associée à l'hémolyse intra vasculaire, aux ulcères de jambe, à une altération de la fonction rénale et hépatique, et à la surcharge en fer. Le mécanisme physiopathologique de l’ HTAP est complexe et hétérogène associant d'une part l'hémolyse à répétition, les troubles du métabolisme du monoxyde d'azote, l'asplénisme fonctionnel, les épisodes de thromboses à répétition et la surcharge en fer, d'autre part la dé-saturation et la maladie pulmonaire chronique sous-jacente. Dans notre travail, cette HTAP était corrélée à des facteurs précédemment identifiés dans la littérature: le type SS d'hémoglobinose [8], le taux d'hémoglobine de base [8], et la dilatation de l'oreillette gauche [15]. D'autres paramètres associés à l'HTAP ont été rapportés: le taux de réticulocytes [8, 30], l'hémolyse avec l’élévation des LDH, de l'acide urique ou de la phosphatase alcaline [9, 26, 27, 29, 30], la ferritinémie [29, 30], le taux de NT-proBNP [30], le taux d’érythropoïétine[26], l'augmentation des globules blancs [28], des plaquettes[25], de l'urée plasmatique [28], l'hypertension artérielle, et la dysfonction diastolique du ventricule gauche et droite [15, 26, 27]. D'autres facteurs sont plus controversés: l’âge, le sexe, les antécédents de syndrome douloureux thoracique, d'accident vasculaire cérébral, de transfusions sanguines et d'un traitement de fond par Hydroxy-urée [25, 26].

## Conclusion

La drépanocytose est une pathologie grave dont le pronostic est aggravé par l'atteinte cardiovasculaire, en particulier l'apparition d'une hypertension artérielle pulmonaire. L’échographie cardiaque est un outil indispensable permettant le dépistage de l'atteinte cardiaque permettant ainsi l'ajustement thérapeutique.
